# MKP-4 suppresses hepatocarcinogenesis by targeting ERK1/2 pathway

**DOI:** 10.1186/s12935-019-0776-3

**Published:** 2019-03-18

**Authors:** Zhongyi Shen, Chengliang Zhang, Lishuai Qu, Cuihua Lu, Mingbing Xiao, Runzhou Ni, Jinxia Liu

**Affiliations:** 1 Department of Gastroenterology, Affiliated Hospital of Nantong University, 20 Xisi Road, Nantong, 226001 Jiangsu People’s Republic of China; 2 Clinical Medicine Medical College, Nantong University, Nantong, Jiangsu People’s Republic of China

**Keywords:** MKP-4, ERK1/2, Hepatocellular carcinoma, Phosphorylation, Prognosis

## Abstract

**Background:**

Mitogen-activated protein kinase phosphatases-4 (MKP-4) is reported to exert a prognostic merit in hepatocarcinogenesis. However, the underlying molecular mechanisms have not been clearly defined.

**Methods:**

Immunoprecipitation-mass spectrometry (IP-MS) approach was used to identify interactive proteins with MKP-4. Western blot and immunohistochemistry were employed to detect proteins in HCC tissues. Cell counting kit-8, colony formation, Edu incorporation and sphere formation assays were performed to investigate functions of MKP-4/ERK1/2 interaction. Tumor xenografts in nude mice were used to determine effects in vivo.

**Results:**

Extracellular signal-regulated kinase 1 and 2 (ERK1/2) were identified as binding partners of MKP-4. Knockdown of MKP-4 increased cell proliferation and cancer stem cell (CSC) traits while upregulation of MKP-4 or pre-incubation with ERK1/2 inhibition reversed these effects. Mechanistically MKP-4 negatively regulated phosphorylation of ERK1/2 and reduced expressions of CyclinD1 and c-Myc. Both xenograft tumor models and clinical analysis of HCC patients indicated that lower expression of MKP-4 and higher expressions of ERK1/2 were associated with worse prognosis.

**Conclusions:**

MKP-4-mediated dephosphorylation of ERK1/2 might serve as a novel tumor-suppressive mechanism and provide a potential therapy for HCC.

## Background

Hepatocellular carcinoma (HCC) is one of the most common cancers and the third leading cause of cancer-related deaths worldwide [1, 2]. Although great progress has been made in therapeutic strategies [3], 5-year survival of HCC is no more than 10% [4, 5]. Identification of molecular mechanisms involved in HCC is of particular significance.

Mitogen-activated protein kinases (MAPKs) are important signal transduction molecules which regulate a variety of cellular processes, including cell differentiation, proliferation and apoptosis [6, 7]. MAPK kinases promote activation of MAPKs by phosphorylation of threonine/serine residues [8]. Dual-specificity phosphatases (DUSPs), which selectively dephosphorylate threonine/serine and tyrosine residues on MAPKs, negatively regulate signal transduction of MAPK cascades [9, 10]. Mitogen-activated protein kinase phosphatases (MKPs) are a subgroup of DUSPs, including 11 members. Recent researches indicated that MKP-4 inhibited the progression of colorectal cancer, gastric cancer and clear cell renal cell carcinoma [11, 12]. It triggers cellular enlargement, microtubule disruption, G2/M-associated cell death, and some features of mitotic catastrophe in epidermal carcinogenesis [13]. Our previous studies revealed MKP-4 as a potential tumor suppressor in hepatocellular carcinoma [14]. However, precise mechanisms remain poorly understood.

In our present study, we used immunoprecipitation-mass spectrometry (IP-MS) analysis and identified extracellular signal-regulated kinases 1 and 2 (ERK1/2) as novel binding partners of MKP-4. ERK1/2 have been recognized as key factors in diverse human cancers, such as lung, bladder, colorectal cancers and so on [15–17]. ERK1/2 are activated and subsequently phosphorylate numerous substrate proteins involved in multiple malignant phenotypic features [18–21]. Dephosphorylation of ERK1/2 on Thr202/Tyr204 or Thr185/Tyr187 residues could inhibit these effects [22, 23]. Is there a potential relationship between MKP-4 and ERK1/2 in HCC incidence?

Here we confirmed that MKP-4 could interact with ERK1/2 and negatively regulate ERK1/2 pathway through dephosphorylating ERK1/2 in liver tumor cells and xenograft tumor models. We also demonstrated that lower expression of MKP-4 was correlated with higher expressions of ERK1/2 and p-ERK1/2 in HCC tissues. On the basis of these findings, we conclude that MKP-4 may suppress hepatocarcinogenesis by targeting ERK1/2 pathway.

## Methods

### Mass spectrometry assay

HCC tissues were drew with immunoprecipitation lysis buffer (25 mM Tris–HCl (pH 7.5), 150 mM NaCl, 1 mM EDTA, and 1% NP-40, pH 7.8) and pre-clarified with protein G Sepharose (Sigma) for 2 h. Protein (100 mg) was immunoprecipited with anti-MKP-4 antibody at 4 °C overnight. The complexes were retrieved with protein G Sepharose for another 2 h. The precipitations were washed three times and then loaded onto 10% polyacrylamide gel and stained with coomassie brilliant blue. The gels were cut and then analyzed for the interacted proteins using an LTQ mass spectrometer (Thermo, San Jose, CA). The peptide maps were clustered and aligned using clustering parameters. The peptide clusters were aligned with Mascot identification files to assign sequence identity. Protein identifications were accepted if they could be established at 95% probability and contained at least two unique identified peptides.

### Western blot and immunoprecipitation analyses

Tissues and cells were promptly homogenized in a lysis buffer containing 50 mM Tris–HCl pH 7.5, 1% Triton X-100, 1% Nonidet P-40, 0.2% sodium dodecyl sulfate (SDS), 0.5% sodium deoxycholate, 1 mM EDTA, 10 μg/ml leupeptin, 10 μg/ml aprotinin, and 1 mM PMSF. The concentrations of protein were determined by a BCA protein assay (Bio-Rad, Hercules, CA, USA). Equal amounts of total proteins were separated by 10% sodium dodecyl sulfate–polyacrylamide gel electrophoresis (SDS-PAGE) and then transferred to a PVDF membrane (Millipore, Bedford, MA). After being blocked in 5% nonfat milk in TBST (20 mM Tris, 150 mM NaCl, 0.05% Tween-20) for 2 h at room temperature, the membranes were incubated overnight at 4 °C with the primary antibodies. Antibodies used were as follows: anti-MKP-4 (1:500, Immunoway, USA); anti-GAPDH (1:5000, Cell Signaling Technology, USA); anti-ERK1/2 (1:500, Santa Cruz Biotechnology, USA); anti-p-ERK1/2 (1:500, Santa Cruz Biotechnology, USA); anti-CyclinD1 (1:1000, Cell Signaling Technology, USA) and anti-c-Myc (1:500, Cell Signaling Technology, USA); followed by incubation with horseradish peroxidase-conjugated secondary human anti-mouse or anti-rabbit antibodies (1:5000, Jackson ImmunoResearch Inc., USA) for 2 h at room temperature. The band was detected by enhanced chemiluminescence detection systems (Cell Signaling Technology, USA) and measured by Image J analysis system (Wayne Rasband, National Institutes of Health USA). For immunoprecipitation, the supernatants of cell lysates or tissues were precipitated with the primary antibodies or control IgG in conjunction with protein G Sepharose. The precipitates were collected for western blot analysis.

### Cell lines, cell culture, plasmid constructs and transfections

Human liver tumor cell lines (HepG2, SK-Hep1 and SMMC-7721), human hepatocyte cell line (LO2) and HEK293 cells were purchased from the Institute of Cell Biology and cultured in Dulbecco modified Eagle’s medium (DMEM; Sigma Chemical) supplemented with 10% fetal bovine serum (FBS; HyClone), penicillin 100 U/ml, and streptomycin 100 μg/ml in an incubator with 5% CO_2_ at 37 °C. The full-length MKP-4 (Gene ID: 1852), MKP-4-siRNAs and MKP-4-shRNA were purchased from GenePharma. The target sequences were as follows: scrambled, 5′-UUCUCCGAACGUGUCACGU-3′; MKP-4-siRNA3, 5′-GUUCUGUCACCGUCACUGU-3′; MKP-4-siRNA4, 5′-CUCUCUCAACGAUGCCUAU-3′; MKP-4-siRNA5, 5′-UCAUGCAGAAGCUCCACCU-3′; MKP-4-siRNA6, 5′-UCAGCAGAUUCCAGGCCGA-3′; MKP-4-shRNA, 5′-UCAUGCAGAAGCUCCACCU-3′. Cell transfections were performed using the Lipofectamine™2000 transfection reagent (Invitrogen) in accordance with manufacturer’s protocol. 48 h after transfection, cells were used for the subsequent experiments. All experiments were repeated at least three times.

### Immunofluorescence assay

Liver tumor cells and sections were firstly blocked with confining liquid consisting of 10% donkey serum, 1% BSA, 0.1% Triton X-100 and 0.05% Tween-20 for 2 h at room temperature to avoid unspecific staining. After that, they were incubated with anti-MKP-4 (anti-mouse, 1:500; Immunoway, USA) and anti-ERK1/2 (anti-rabbit, 1:500; Santa Cruz Biotechnology) at 4 °C overnight. A mixture of FITC- and TRITC-conjugated secondary antibodies were added and incubated for 2 h at room temperature. Finally, cells and sections were examined with a Leica fluorescence microscope (Leica, DM 5000B, Leica CTR 5000, Germany).

### Cell counting kit-8 assay

Cell proliferation assay was performed by cell counting kit-8 (CCK-8) solution according to the manufacturer’s protocol. Liver tumor cells were firstly plated at a density of 2 × 10^4^ cells per well in 100 μl volume in a 96-well plate. Then cells were incubated with 90 μl complete DMEM medium and 10 μl CCK-8 reagent (Dojindo, Kumamoto, Japan) under different treatments after cell adherence. Cells were incubated for 2 h at 37 °C and the absorbance was measured at 490 nm and 630 nm using a microplate reader (Bio-Rad).

### Colony formation assays

For colony formation assays, liver tumor cells (500 cells per well) were plated in 6-well culture plates. After 2 weeks, the surviving colonies (50 cells per colony) were counted after staining with 0.5% crystal violet for 30 min.

### Edu incorporation assay

Cells were plated into a 96-well plate and then labeled with 20 μM Edu overnight. After labeling and washing, cells were fixed with formaldehyde rinsed and stained with Alexa488-azide for 20 min. After washing three times with PBS with 0.5% Triton X-100, the cells were stained with 10 μM Hoechst 33,342 for 30 min. The cells were washed again and imaged by fluorescence microscopy.

### Sphere formation assay

HepG2 and SK-Hep1 cells were incubated in anchorage-independent conditions for tumor sphere formation assay. Liver tumor cells were seeded into 6-well plates and maintained in serum-free medium. Basic fibroblast growth factor (b-FGF; 10 ng/ml; R&D Systems) and fresh epidermal growth factor (EGF; 20 ng/ml; R&D Systems) were added every other day. The radius of each tumor spheroid and the number of tumor spheres were was measured using NIS-Elements Microscope Imaging Software (Nikon, Tokyo, Japan) after 2 weeks.

### Establishment of stable expression cell lines

For lentivirus production, 1 μg of Myc-tagged MKP-4 and MKP-4-shRNA plasmids together with 1 μg of helper plasmids (0.4 μg pMD2G and 0.6 μg psPAX2) were transfected into HEK293T cells with effectene reagent (Qiagen, Valencia, CA, USA). Viral supernatants were collected 48 h after transfections and cleared through a 0.45-μm filter. HepG2 cells were infected with the virus and selected with 1 mg/ml puromycin (Sigma) to get stable MKP-4-expressing and MKP-4-knockdown cells.

### Xenograft mouse model

Five-week-old female nude mice purchased from Shanghai SLAC Animal Center were raised in a pathogen-free condition. A total of 2 × 10^6^ HepG2-shMKP-4; HepG2-MKP-4 or HepG2-control cells were re-suspended in 200 μl PBS and injected subcutaneously into the nude mice. The tumor volume was measured for 7 days with a vernier caliper and calculated on the basis of the following formula: volume (mm^3^) = length × width × height × 0.52.) [24]. The mice were sacrificed 28 days after injection and the tumors were removed and weighted. The experimental protocol was approved by the Committee on Animals Care and Use of Nantong University.

### Patients and tissues

A panel of formalin-fixed, paraffin-embedded HCC and corresponding para-cancerous tissues were obtained from 160 patients diagnosed with HCC at the Affiliated Hospital of Nantong University from 2006 to 2010. None of the patients received preoperative interventional therapy or systemic chemotherapy. The main clinical and pathological features (including age, gender, tumor size, differentiation, 5-year follow-up survival records and other information) showed in Table 1 were obtained from the medical records. The patients included 125 males and 35 females with an average age of 51.84 years (range from 32 to 71 years). Tumor differentiation was assessed by Edmondson grading system. Liver function was assessed by Child–Pugh-classification. All the patients were typed in accordance with the sixth edition of tumor-node-metastasis classification (TNM). Overall survival was defined as the interval between surgery and death or the last follow-up appointment. The study was approved by the Ethical Research Committee of the Affiliated Hospital of Nantong University.

**Table 1:** Association of MKP-4 expression, ERK1/2 expression and p-ERK1/2 expression with clinicopathological parameters in 160 hepatocellular carcinoma specimens

Parameters	Total	MKP-4 expression		*P*	ERK1/2 expression		*P*	p-ERK1/2 expression		*P*
		Low	High		Low	High		Low	High	
Age										
≤ 45	82	66	16	0.051	24	58	0.060	36	46	0.902
> 45	78	51	27		34	44		35	43	
Gender										
Female	35	25	10	0.798	13	22	0.901	13	22	0.330
Male	125	92	33		45	80		58	67	
Tumor differentiation										
I–II	78	46	32	< 0.001*	37	41	0.004*	41	37	0.042*
III–IV	82	71	11		21	61		30	52	
Tumor size										
≤ 5	92	67	25	0.921	30	62	0.265	42	50	0.705
> 5	68	50	18		28	40		29	39	
HBsAg										
Negative	31	25	6	0.293	11	20	0.921	14	17	0.922
Positive	129	92	37		47	82		57	72	
Liver cirrhosis										
Negative	49	39	10	0.220	14	35	0.179	23	26	0.665
Positive	111	78	33		44	47		48	63	
Tumor encapsulation										
None	60	44	16	0.963	21	39	0.799	31	29	0.150
Complete	100	73	27		37	63		40	60	
Child–Pugh score										
A	77	58	19	0.545	31	44	0.310	36	41	0.560
B	83	59	24		27	56		35	48	
Microvascular invasion										
Negative	65	40	25	0.006*	30	35	0.031*	28	37	0.785
Positive	95	77	18		28	67		43	52	
AFP (ng/ml)										
≤ 50	122	93	29	0.112	40	82	0.103	52	70	0.424
> 50	38	24	14		18	20		19	19	
TNM stage										
I–II	84	54	30	0.008*	38	46	0.013*	45	39	0.014*
III–IV	76	63	13		20	56		26	50	
Tumor number										
Single	78	60	18	0.504	27	51	0.675	38	46	0.902
Multiple	82	57	25		31	51		20	56	

Statistical analyses were performed by the Pearson χ^2^ test

* *P *< 0.05 was considered significant

### Immunohistochemistry

For immunohistochemically analysis, HCC sections were deparaffinized and rehydrated with graded ethanol, then soaked in EDTA (1 mmol/L, pH 8.0) and heated to 121 °C to retrieve the antigen. After rinsing with phosphate-buffered saline (PBS, pH 7.2), 0.3% Hydrogen peroxide was applied to block endogenous peroxide activity for 20 min, 10% goat serum was applied to block any nonspecific reactions for 1 h. After washing with PBS, the sections were incubated with the primary antibody overnight. All sections were processed using the peroxidase-anti-per-oxidase method (Dako, Hamburg, Germany). The slides were counterstained with DAB (0.1% phosphate buffer solution, 0.02% diaminobenzidine tetrahydrochloride, and 3% H_2_O_2_) dehydrated, and fastened with resin mount. Finally, the slides were examined with a Leica CTR5000 microscope (Leica Microsystems, Wetzlar, Germany).

### Statistical analysis

All statistical analyses were performed using SPSS (Statistical Product and Service Solutions) 20.0 software package. Statistical analyses of continuous variables were performed by Student’s *t* test. Paired t-tests were used to compare xenograft tumor size and MKP-4 expressions in paired clinical samples. Pearson’s Chi square test was performed to evaluate associations between MKP-4, ERK1/2 and p-ERK1/2 expressions and clinicopathological factors. Kaplan–Meier plots and log-rank tests were used for overall survival analysis. Multivariate analysis was constructed using the Cox proportional hazards model. *P *< 0.05 was considered statistically significant. All statistical tests were two-sided.

## Results

### Interaction between MKP-4 and ERK1/2 in HCC

**Fig. 1:**
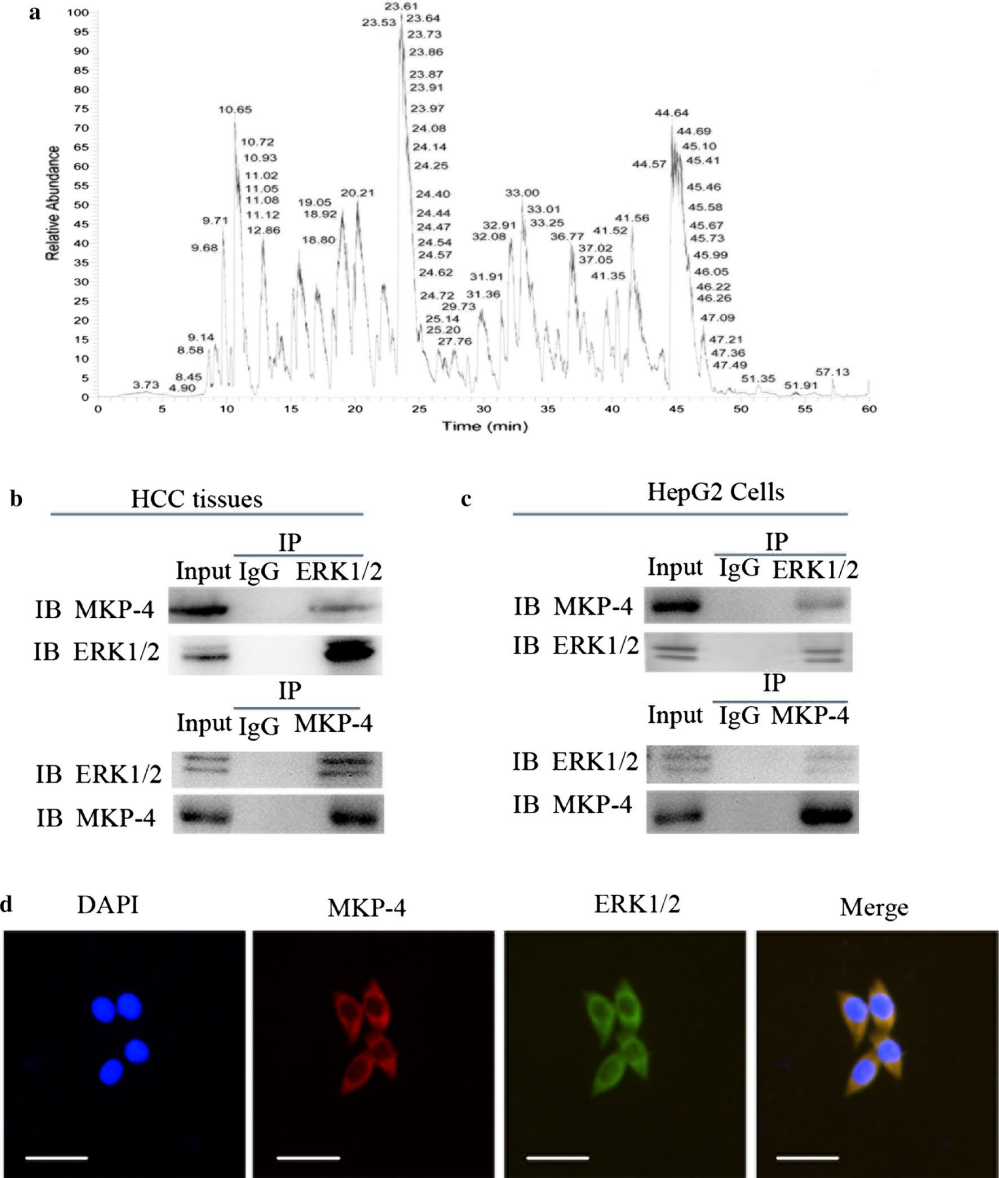
MKP-4 interacts with ERK1/2 in liver tumor cells and tissues. **a** The results of mass spectrometry in HCC tissues. **b** Verification of the interaction between MKP-4 and ERK1/2 in HCC tissues using immunoprecipitation assay. **c** Reciprocal immunoprecipitation of MKP-4 and ERK1/2 in HepG2 cells. Lysates of HepG2 cells were immunoprecipitated with anti-MKP-4, anti-ERK1/2 antibodies or control IgG. The immunoprecipitates were subjected to western blot analysis with anti-ERK1/2 and anti-MKP-4 antibodies. **d** Immunofluorescence analysis of MKP-4 and ERK1/2 in HepG2 cells. HepG2 cells were subjected to immunofluorescence assay using anti-MKP-4 and anti-ERK1/2 antibodies. Scale bar: 50 μm

ERK1/2 were identified as novel binding partners of MKP-4 in HCC tissues (Fig. 1a). Immunoprecipitation assay was performed in HCC tissues and HepG2 cells to validate the interaction of MKP-4/ERK1/2 (Fig. 1b, c). In addition, immunofluorescence staining revealed that MKP-4 and ERK1/2 proteins were co-localized in cytoplasm of HepG2 cells (Fig. 1d), which provided further support for a functional interplay.

### MKP-4 regulates phosphorylation of ERK1/2 in liver tumor cells

**Fig. 2:**
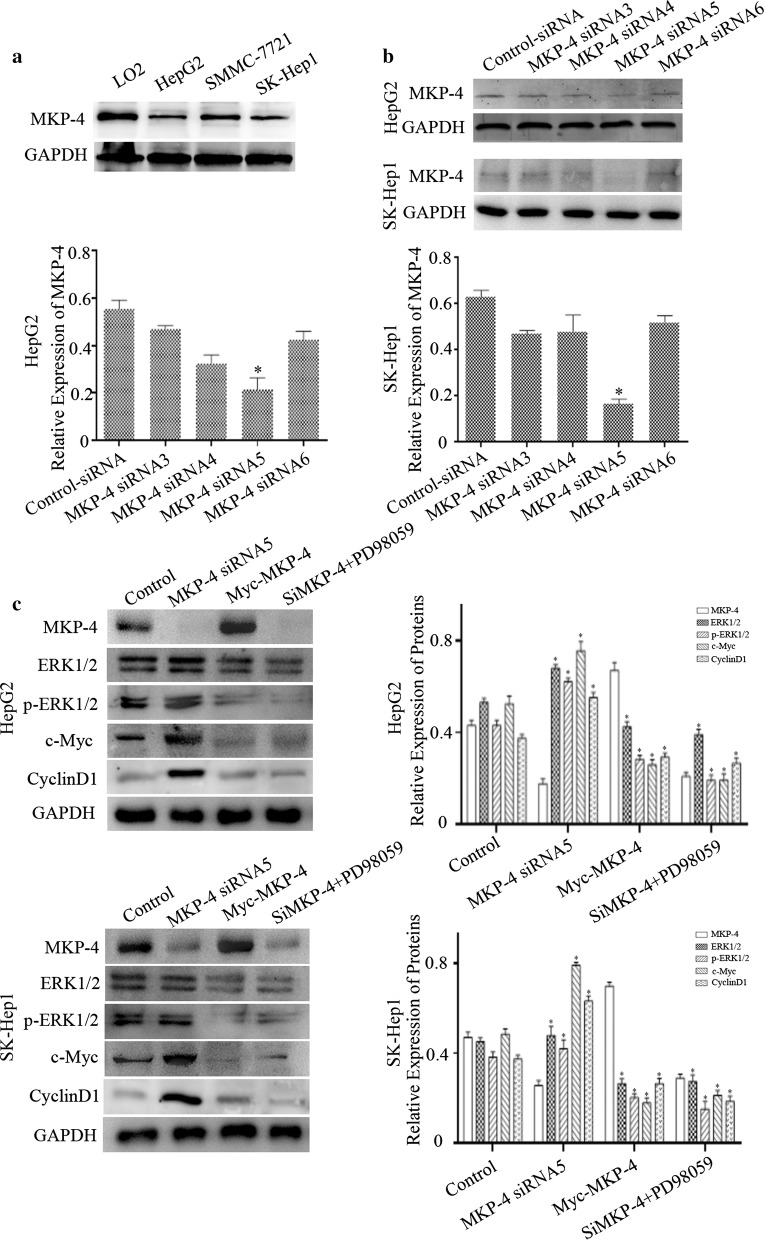
MKP-4 regulates the phosphorylation of ERK1/2 in liver tumor cells. **a** The expressions of MKP-4 in LO2 and different liver tumor cells were detected by using western blot. **b** We used RNA interference to knockdown MKP-4 expression in HepG2 or SK-Hep1 cells and chose best interfering efficiency. The bar chart demonstrated the ratio of MKP-4 expression to GAPDH by densitometry. The data were mean ± SEM of three independent experiments (*P < 0.05). **c** The expressions of ERK1/2, p-ERK1/2 and the downstream target genes in different treated cells. The bar chart showed the ratio of these proteins to GAPDH by densitometry. The data were mean ± SEM of three independent experiments. (*P < 0.05)

Since p-ERK1/2 is the active form of ERK1/2 and plays a vital role in tumor progression, we speculated whether MKP-4 could regulate ERK1/2 phosphorylation. We employed western blot analysis and found that MKP-4 expression was obviously down-regulated in liver tumor cells, as compared with LO2 hepatocytes (Fig. 2a). We used RNA interference to knockdown MKP-4 expression in HepG2 or SK-Hep1 cells and found that MKP-4 siRNA5 exerted the best interfering efficiency (Fig. 2b). Moreover, Myc-tagged MKP-4 was employed to upregulate MKP-4 expression in liver tumor cells. After that, we detected the expressions of p-ERK1/2 and downstream genes in different treated cells as shown in Fig. 2c. The results indicated that expressions of p-ERK1/2, CyclinD1 and c-Myc were decreased by overexpression of MKP-4 or pre-incubation of 10 μM PD98059 by 24 h while expressions of the above genes were increased by MKP-4 interference. These data suggested that MKP-4 could regulate phosphorylation of ERK1/2 and ERK1/2 pathway in liver tumor cells.

### MKP-4 inhibits cell proliferation and cancer stem cell (CSC) traits through ERK1/2 pathway

**Fig. 3:**
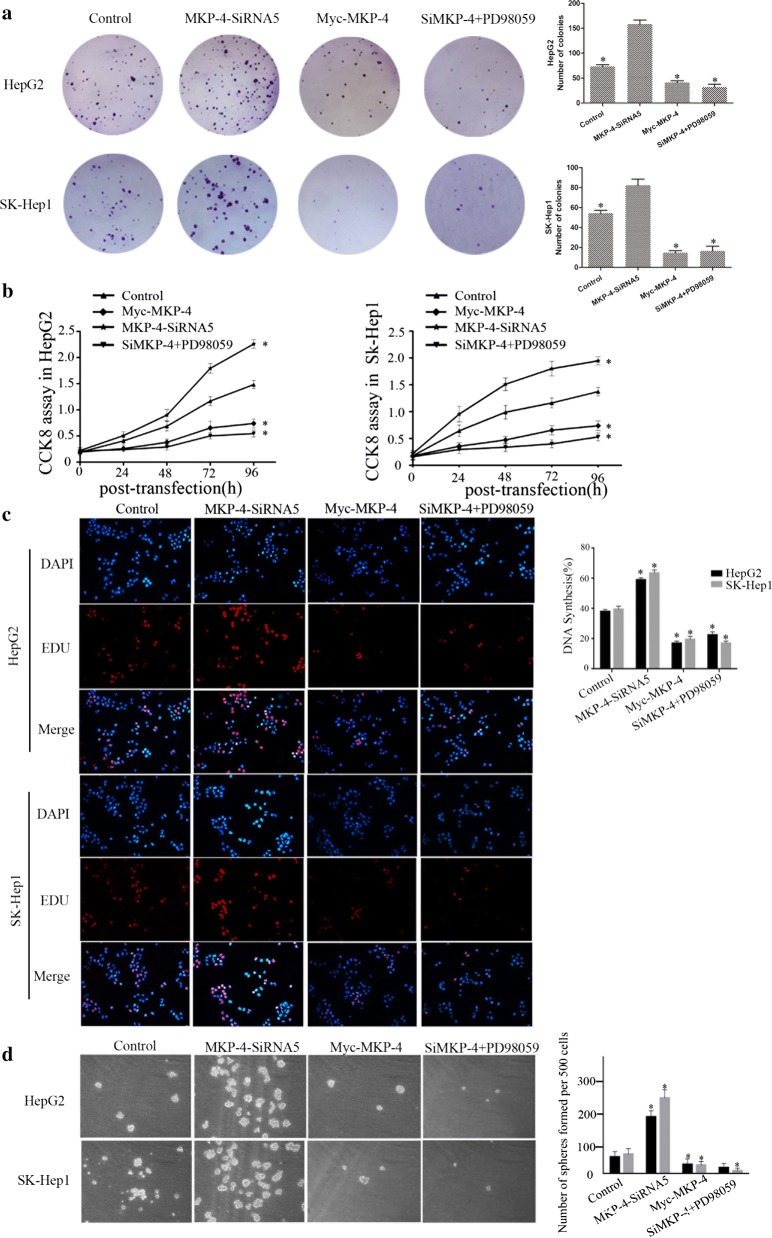
MKP-4 inhibits cell proliferation and cancer stem cell (CSC) traits through ERK1/2 pathway. **a** MKP-4 reduced the colony formation of HepG2 or SK-Hep1 cells via the interaction with ERK1/2. For colony formation assay, MKP-4 knockdown, MKP-4 overexpression or pre-incubation with 10 μM PD98059 by 24 h cells were seeded into each well of six-well-plate colonies and stained with crystal violet after 2 weeks. **b** CCK-8 assay showed that overexpression of MKP-4 or pre-incubation with 10 μM PD98059 by 24 h inhibited cell proliferation in HepG2 and SK-Hep1 cells while MKP-4 depletion promoted cell proliferation. The data are mean ± SEM of three independent experiments. (*P < 0.05, compared with the control group). **c** MKP-4 inhibited DNA synthesis via the interaction with ERK1/2 by using the Click-iT Edu Alexa Fluor Imaging Kit in HepG2 and SK-Hep1 cells. The bar chart demonstrated the DNA synthesis of liver tumor cells. The data were mean ± SEM of three independent experiments (*P < 0.05, compared with the control group). **d** The interaction of MKP-4 and ERK1/2 plays an essential role in sphere formation. Downregulation of MKP-4 promoted sphere formation ability while overexpression of MKP-4 or pre-incubation with 10 μM PD98059 by 24 h inhibited the ability. The bar chart showed the number of sphere formation per 500 cells. The data were mean ± SEM of three independent experiments (*P < 0.05, compared with control group)

We investigated biological effects of the interaction between MKP-4 and ERK1/2 following different treatments. Colony formation, CCK-8 assays and Edu assays indicated that proliferation of HepG2 and SK-Hep1 cells were significantly increased after MKP-4 depletion, whereas overexpression of MKP-4 impaired the capacity of cell proliferation. Moreover, treatment with 10 μM PD98059, an antagonist of ERK kinases by 24 h, abrogated the pro-proliferative effect of MKP-4 depletion in liver tumor cells (Fig. 3a–c). CSCs can form spheres in the absence of serum under low adherence conditions. Therefore, we evaluated the ability of different treated cells to grow spheres under serum-free conditions. The ability of tumor sphere formation was decreased in MKP-4-overexpressing cells or MKP-4-lacking cells with the inhibition of ERK1/2 pathway whereas the ability was enhanced accompanied by the decreased expression of MKP-4 (Fig. 3d).

### MKP-4 suppresses tumor growth in vivo through the modulation of ERK1/2 signaling

**Fig. 4:**
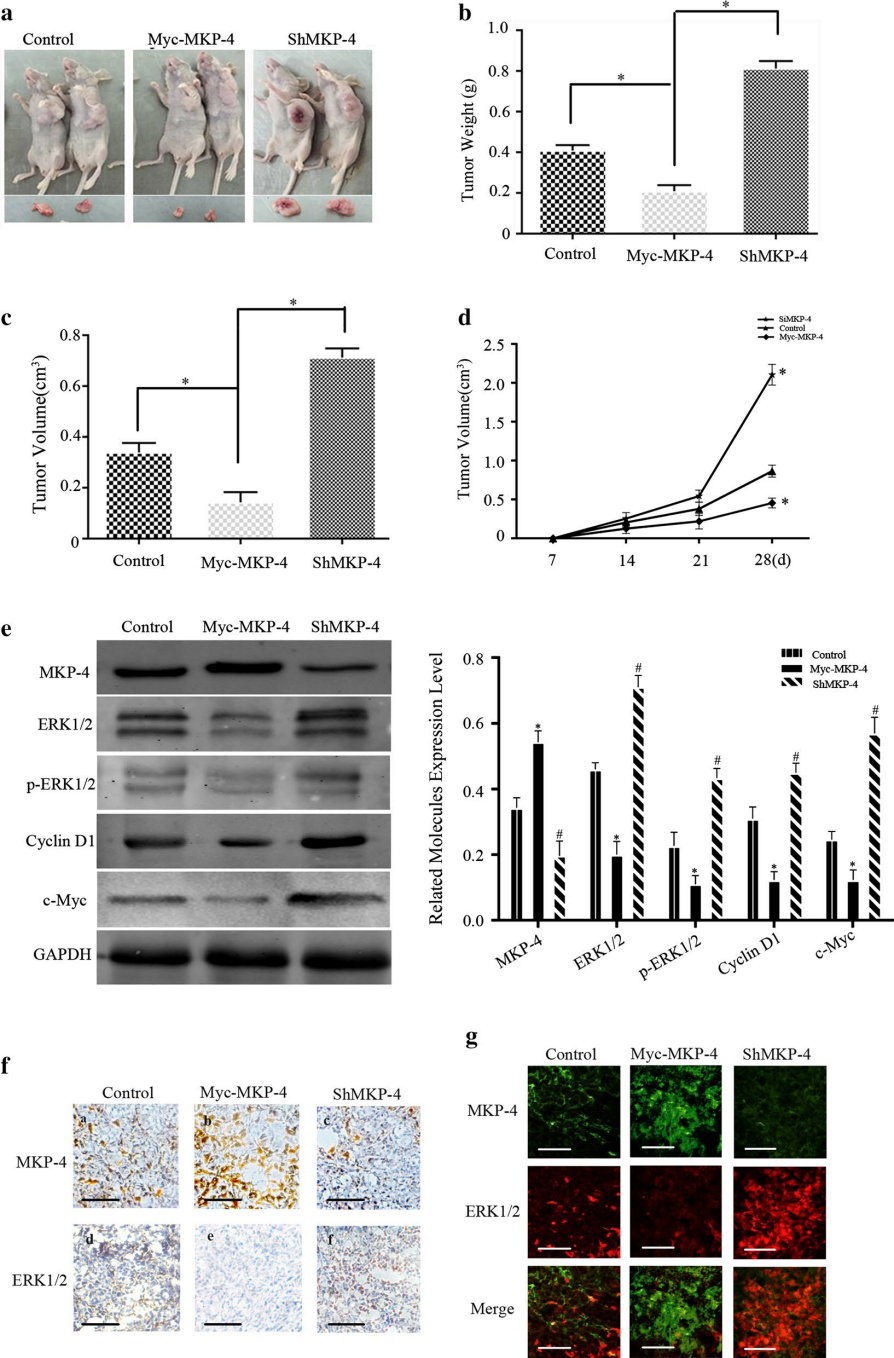
MKP-4 inhibits the tumorigenicity of HCC by targeting ERK1/2 pathway in vivo. **a** MKP-4 inhibits tumor growth of HepG2 cells in vivo. Control, MKP-4 silenced or MKP-4 overexpressed HepG2 cells were injected into BALB/c nude mice. **b**, **c** Tumor weight and volume harvested from the nude mice in different groups after 4 weeks. *P < 0.01, referring to differences between different groups. **d** The silence of MKP-4 significantly promotes tumor growth while MKP-4 overexpression inhibited tumor growth in nude mice. The data are mean ± SEM of three independent experiments. **e** Lysates from resected tumor specimens were used to detect the expression of ERK1/2, p-ERK1/2 and downstream targets of ERK1/2 pathway. The bar chart of the relative protein expressions in tumor tissues under the indicated treatments. The data are mean ± SEM (*P < 0.05). **f** Immunohistochemical analysis of MKP-4 and ERK1/2 in MKP-4-overexpressing or silenced tumors. **g** Immunofluorescent analysis of MKP-4 and ERK1/2 in MKP-4-overexpressing or silenced tumors. Scale bar: 100 μm

We then examined the effect of MKP-4 on HCC progression using subcutaneous xenograft model. As shown in Fig. 4a, tumors in HepG2-MKP-4 group grew much slower than HepG2-control and HepG2-shMKP-4 groups. Both tumor volumes and weights in HepG2-MKP-4 group were significantly lower than the other two groups 28 days after the subcutaneous implantation (Fig. 4b, c). The volumes of MKP-4-overexpressing tumors increased slower than other groups, as indicated by tumor growth curves (Fig. 4d). These results indicated that depletion of MKP-4 significantly promoted the progression of HCC in vivo. To further clarify whether the interaction between MKP-4 and ERK1/2 is involved in tumor progression, we detected the expressions of MKP-4, ERK1/2 and p-ERK1/2 using western blot and immunohistochemistry analyses. The results showed that MKP-4 significantly decreased the phosphorylation levels of ERK1/2 (Fig. 4e–g). Together, these findings implicated that MKP-4 suppresses growth of HCC in nude mice via regulation of ERK1/2 pathway.

### Expressions of MKP-4, ERK1/2 and p-ERK1/2 in HCC tissues

**Fig. 5:**
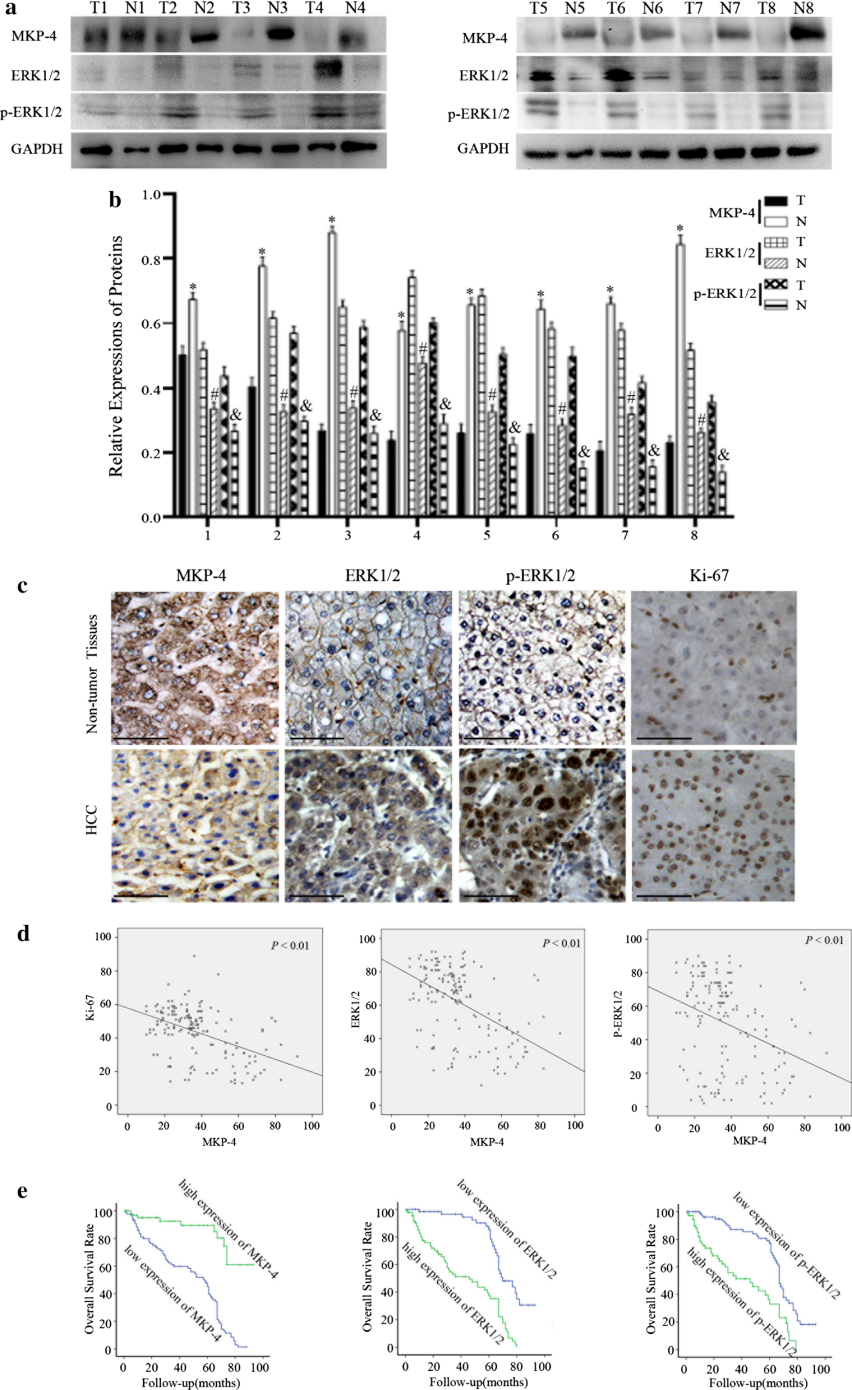
Expressions and prognostic roles of MKP-4/ERK1/2 in HCC patients. **a** Western blot analysis revealed a lower expression of MKP-4 or higher expressions of ERK1/2, p-ERK1/2 in hepatocellular carcinoma (T) and adjacent non-tumorous tissues (N). **b** The bar chart demonstrates the ratio of MKP-4, ERK1/2 and p-ERK1/2 to GAPDH by quantitative analysis. *P < 0.05 compared with adjacent normal liver tissue. **c** Immunohistochemical analysis of MKP-4, ERK1/2 p-ERK1/2 and Ki-67 expressions in paraffin-embedded tissue sections. Scale bar: 100 μm. **d** Relationship between MKP-4, ERK1/2, p-ERK1/2 and Ki-67 expression in HCC patients. Scatter plot of them with regression line showing a significant correlation using the Pearson’s test (*P *< 0.01). **e** Kaplan–Meier survival curve according to MKP-4, p-ERK1/2 and ERK1/2 in 160 HCC patients (*P* < 0.05, log-rank test)

To further determine the relationship between MKP-4, ERK1/2 and p-ERK1/2, we analyzed the expressions in eight paired HCC and adjacent non-tumorous tissues using western blot analysis. Our results revealed significantly lower expression of MKP-4 and higher expression of ERK1/2, p-ERK1/2 in HCC tissues than in the non-tumorous tissues (Fig. 5a, b). Furthermore, we performed immunohistochemical analysis to detect the expression of MKP-4, ERK1/2, p-ERK1/2 and Ki-67 in 160 HCC specimens and found that expression of MKP-4 was frequently downregulated while the expressions of ERK1/2, p-ERK1/2 and Ki-67 were elevated in tumorous samples compared with non-tumorous tissues (Fig. 5c, d).

### Relationship between expressions of MKP-4, ERK1/2, p-ERK1/2 and clinicopathological factors of HCC

To reveal the correlation between protein expressions and clinical characteristics, clinical samples were divided into low and high expression groups according to immunohistochemical evaluation. As shown in Table 1, expressions of MKP-4, ERK1/2 and p-ERK1/2 were correlated with tumor differentiation (*P *< 0.001, *P *= 0.004 and *P *= 0.042), microvascular invasion (*P *= 0.006, *P *= 0.031 and *P *= 0.078) and TNM stage (*P *= 0.008, *P *= 0.013 and *P *= 0.014). However, there was no significant relationship between other prognostic factors, such as age, gender, tumor size, tumor number, Child–Pugh score, tumor encapsulation, HBsAg and serum AFP level. Furthermore, univariate analysis showed that tumor differentiation, TNM stage, MKP-4 expression, ERK1/2 expression and p-ERK1/2 expression were significantly associated with patients’ survival (Tables 2, 3). In addition, further studies showed that MKP-4 expression was positively correlated with ERK1/2 and p-ERK1/2 expression in HCC tissues (Table 4). Kaplan–Meier survival curves indicated that low expression of MKP-4 and high expression of ERK1/2, p-ERK1/2 were significantly associated with poor overall survival (Fig. 5e).

**Table 2:** Univariate of factors associated with overall survival (n = 160)

Variables	Hazard ratio	95% confidence interval	*P* value
Age (> 45 vs ≤ 45)	0.739	0.437–1.249	0.258
Gender (male vs female)	0.995	0.561–1.764	0.986
Tumor differentiation (III–IV vs I–II)	2.267	1.339–3.837	0.002*
Tumor size, cm (> 5 vs ≤ 5)	0.997	0.587–1.694	0.991
HBsAg (positive vs negative)	0.865	0.461–1.624	0.652
Liver cirrhosis (positive vs negative)	1.025	0.619–1.695	0.925
Tumor encapsulation (none vs complete)	1.155	0.704–1.893	0.568
Child–Pugh score (B vs A)	0.767	0.466–1.260	0.295
Microvascular invasion (positive vs negative)	1.852	1.019–3.368	0.043*
AFP, ng/ml (> 50 vs ≤ 50)	1.431	0.730–2.803	0.297
TNM stage (III–IV vs I–II)	0.549	0.319–0.945	0.030*
Tumor number (multiple vs single)	0.690	0.403–1.180	0.175
MKP-4 (low vs high)	0.145	0.069–0.304	< 0.001*
ERK1/2 (low vs high)	2.377	1.226–4.610	0.010*
p-ERK1/2 (low vs high)	2.874	1.500–5.507	0.001*

Statistical analyses were performed using log-rank test

* *P *< 0.05 was considered significant

**Table 3:** Multivariate analyses of factors associated with overall survival (n = 160)

Variables	Hazard ratio	95% confidence interval	P value
Age (> 45 vs ≤ 45)	0.754	0.460–1.237	0.264
Gender (male vs female)	1.084	0.618–1.900	0.779
Tumor differentiation (III–IV vs I–II)	1.939	1.204–3.120	0.006*
Microvascular invasion (positive vs negative)	1.688	0.985–2.894	0.057
TNM stage (III–IV vs I–II)	0.585	0.357–0.960	0.034*
MKP-4 (low vs high)	0.175	0.086–0.355	< 0.001*
ERK1/2 (low vs high)	2.120	1.137–3.953	0.018*
p-ERK1/2 (low vs high)	2.482	1.375–4.482	0.003*

Statistical analyses were performed using log-rank test

* P < 0.05 was considered significant

**Table 4:** The correlation between MKP-4 expression, ERK1/2 expression and p-ERK1/2 expression in 160 hepatocellular carcinoma specimens

MKP-4 expression	ERK1/2 expression		*P*	p-ERK1/2 expression		*P*
	Low	High		Low	High	
Low	20	97	< 0.001*	46	71	0.034*
High	38	5		25	18	

Statistical analyses were performed using spearman’s rank correlation test

* *P *< 0.05 was considered significant

## Discussion

Hepatocellular carcinoma, especially diagnosed at an advanced stage, has been considered to be one of the most fatal cancers [25]. Novel molecular targets for diagnosis and therapy of HCC are urgently needed to improve HCC prognosis. Recently, deregulation of MAPK pathways has been identified to play a vital role in the pathogenesis of HCC [26–28]. Therefore, it is vital to seek potential mechanisms underlying deregulation of MAPK pathways in HCC initiation and progression.

MAPK pathways, highly conserved in the majority of eukaryotes, play key roles in cellular developmental and physiological processes by delivering extracellular signals into nuclei [29]. Aberrant activation of MAPK pathways are reported to be associated with development of tumors [30]. Undergoing a cascade of sequential phosphorylation events mediated by upstream MEK kinases, phosphorylation of MAPKs on threonine and tyrosine residues can be activated [31]. MKP-4 is a member of MAPK phosphatases which is composed of two domains, MAPK-binding domain in N-terminal whereas the dual-specificity phosphatase domain in C-terminal [32]. ERK1/2 are critical members of MAPKs and involved in plenty of fundamental cellular processes by regulating the phosphorylation of various substrates [33]. In our study, we detected interaction and explored functions of MKP-4/ERK1/2 in HCC both in vivo and in vitro. We speculates that association of MKP-4 to ERK1/2 is MBP-dependent through direct binding of the two proteins and this will be further confirmed by truncation analysis or GST pull-down. ERK1/2 can translocate into nucleus and promote transcription by phosphorylation in HCC, while combination of MKP-4 and ERK1/2 greatly reduces the entry of p-ERK1/2. This result is consistent with MKP-1, which is downregulated and controls ERK1/2 phosphorylation in HCC [34, 35]. Interestingly, evidence here showed that MKP-4 also negatively regulates total protein level of ERK1/2. MKP-4 may affects protein stability of ERK1/2 followed by effects on phosphor-dynamics of ERK1/2. We will perform additional experiments regarding the protein stability of ERK1/2 by MKP-4 in further study.

A small subset within tumour bulk which was defined as tumour-initiating cells (TICs), are considered to be source of tumors including HCC [36]. Liver TICs are reported to be responsible for tumorigenesis and intervention of TIC self-renewal can be a potential treatment in HCC [37]. To determine effect of MKP-4/ERK1/2 interaction in self-renewal potential of liver TICs, we performed sphere formation and validated the promotion of liver TICs self-renewal by MKP-4/ERK1/2 interaction. Since c-Myc which has been recognized as a vital regulator of stem cell biology can serve as a link connecting malignancy and stem cells [38], we detected its expression in different treated cells and tissues from xenograft mice. Our results demonstrated that depletion of MKP-4 increased c-Myc expression, while overexpression of MKP-4 decreased its expression. In consequence, we speculated that interaction of MKP-4 and ERK1/2 inhibit self-renew of liver tumor cells and HCC initiation partly through the transcription factor c-Myc which is a downstream target gene of ERK1/2 pathway. Although lots of transcription factors and signal pathways have been reported to participate in stem cell self-renewal. Due to limitation of time and money, we have not carried out a systematic and comprehensive study in this aspect and just found such a phenomenon. We will do further study in the future.

Our results demonstrate that MKP-4 was downregulated in HCC and that lower expressions of MKP-4 were closely related to higher expressions of ERK1/2 and p-ERK1/2, which are indicators of poor prognosis in HCC. DNA methylation of promoter-associated CpG islands can function as a potential mechanism of silencing tumor suppressor genes in numerous cancers, including HCC [39]. Hypermethylation of CpG islands in the promoter region of tumor suppressor genes is a major event in the development of many cancers [40]. MKP-4 also acts as a tumor suppressor gene in many other cancers in addition to HCC and it has been reported that promoter methylation of DUSP9 in human gastric cancer and colorectal cancer is an important reason for its decreased expression [11, 12]. This may be one of the reason for decreased expression of MKP-4 in HCC.

## Conclusion

**Fig. 6:**
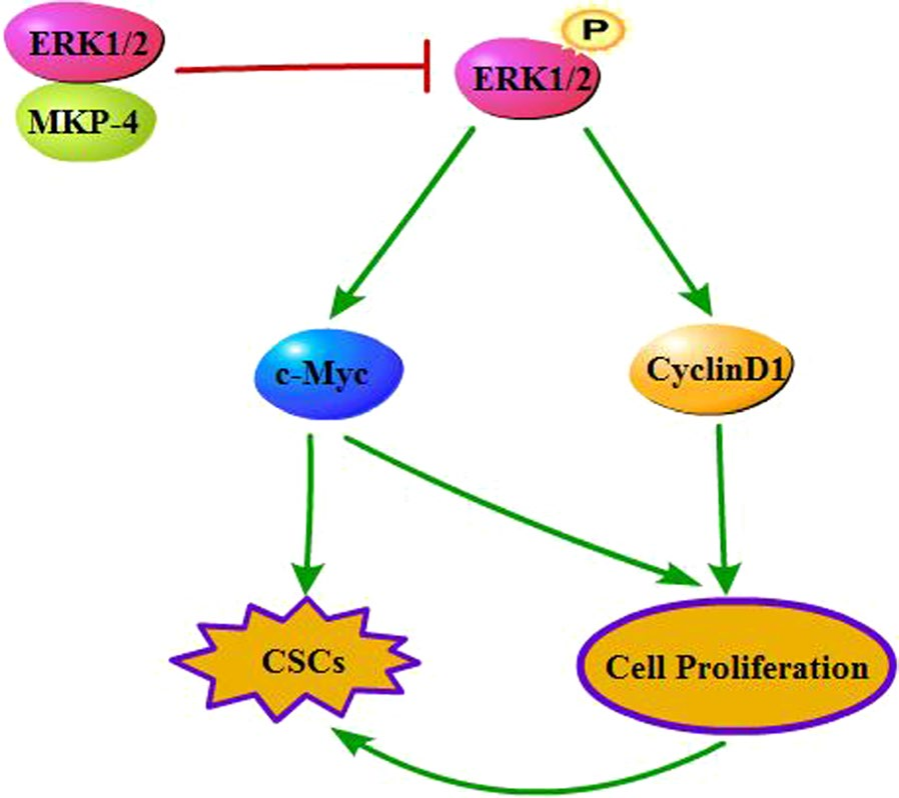
Schematic diagram of proposed mechanism. MKP-4 expression may regulate hepatocellular carcinoma cells proliferation and stemness by inhibiting the phosphorylation of ERK1/2 and enhancing expression of CyclinD1 and c-Myc

We demonstrate that MKP-4 inhibits the occurrence and development of HCC through directly promoting the dephosphorylation of ERK1/2 and decreasing expression of CyclinD1 and c-Myc (Fig. 6). Thus, we supposed the dephosphorylation of ERK1/2 by MKP-4 may act as a promising therapeutic strategy in HCC.

## Abbreviations

HCC: Hepatocellular carcinoma
ERK1/2: extracellular signal-regulated kinase 1 and 2
IP-MS: immunoprecipitation-mass spectrometry
CSC: cancer stem cell
MAPKs: mitogen-activated protein kinases
DUSPs: dual-specificity phosphatases
MKPs: mitogen-activated protein kinase phosphatases
SDS: sodium dodecyl sulfate
SDS-PAGE: sodium dodecyl sulfate–polyacrylamide gel electrophoresis
DMEM: Dulbecco modified Eagle’s medium
FBS: fetal bovine serum
CCK-8: cell counting kit-8
b-FGF: basic fibroblast growth factor
EGF: epidermal growth factor
TNM: tumor-node-metastasis classification

## References

[1] Rapti I, Hadziyannis S (2015). Risk for hepatocellular carcinoma in the course of chronic hepatitis B virus infection and the protective effect of therapy with nucleos(t)ide analogues. World J Hepatol..

[2] Chen W (2016). Cancer statistics in China, 2015. CA Cancer J Clin.

[3] Sparchez Z (2016). Contrast enhanced ultrasonography in assessing the treatment response to transarterial chemoembolization in patients with hepatocellular carcinoma. Med Ultrason..

[4] Hafeez Bhatti AB, Dar FS, Waheed A, Shafique K, Sultan F, Shah NH (2016). Hepatocellular Carcinoma in Pakistan: national trends and global perspective. Gastroenterol Res Pract..

[5] Liu P (2015). Exploring the molecular mechanism and biomakers of liver cancer based on gene expression microarray. Pathol Oncol Res..

[6] Wu GS (2007). Role of mitogen-activated protein kinase phosphatases (MKPs) in cancer. Cancer Metastasis Rev.

[7] Lee M (2013). Mitogen-activated protein kinase phosphatase-1 inhibition and sustained extracellular signal-regulated kinase 1/2 activation in camptothecin-induced human colon cancer cell death. Cancer Biol Ther.

[8] Rojo F (2009). Mitogen-activated protein kinase phosphatase-1 in human breast cancer independently predicts prognosis and is repressed by doxorubicin. Clin Cancer Res.

[9] Boulding T, Wu F, McCuaig R, Dunn J, Sutton CR, Hardy K (2016). Differential roles for DUSP family members in epithelial-to-mesenchymal transition and cancer stem cell regulation in breast cancer. PLoS ONE.

[10] Muda M (1997). Molecular cloning and functional characterization of a novel mitogen-activated protein kinase phosphatase, MKP-4. J Biol Chem.

[11] Jenner S (2015). Development of a DUSP9 methylation screening assay. Pathol Oncol Res..

[12] Wu F (2015). Epigenetic silencing of DUSP9 induces the proliferation of human gastric cancer by activating JNK signaling. Oncol Rep.

[13] Wu S (2011). Decreased expression of dual-specificity phosphatase 9 is associated with poor prognosis in clear cell renal cell carcinoma. BMC Cancer..

[14] Liu J (2013). Decreased expression and prognostic role of mitogen-activated protein kinase phosphatase 4 in hepatocellular carcinoma. J Gastrointest Surg..

[15] Wang Z (2016). Cordycepin induces apoptosis and inhibits proliferation of human lung cancer cell line H1975 via inhibiting the phosphorylation of EGFR. Molecules..

[16] Muhammad N (2016). Anti-miR-203 suppresses breast cancer growth and stemness by targeting SOCS3. Oncotarget..

[17] Okabe H (2006). A critical role for FBXW8 and MAPK in cyclin D1 degradation and cancer cell proliferation. PLoS ONE.

[18] Wang Y (2016). Visfatin stimulates endometrial cancer cell proliferation via activation of PI3 K/Akt and MAPK/ERK1/2 signalling pathways. Gynecol Oncol.

[19] Persaud SD (2016). Corrigendum: all trans-retinoic acid analogs promote cancer cell apoptosis through non-genomic Crabp1 mediating ERK1/2 phosphorylation. Sci Rep..

[20] Xu Y (2016). The antitumor effect of TIG3 in liver cancer cells is involved in ERK1/2 inhibition. Tumour Biol.

[21] Liao YJ (2015). Niemann-Pick type C2 protein regulates liver cancer progression via modulating ERK1/2 pathway: clinicopathological correlations and therapeutical implications. Int J Cancer.

[22] Steelman LS (2001). Roles of the Raf/MEK/ERK and PI3K/PTEN/Akt/mTOR pathways in controlling growth and sensitivity to therapy-implications for cancer and aging. Aging (Albany NY)..

[23] Chang F (2003). Signal transduction mediated by the Ras/Raf/MEK/ERK pathway from cytokine receptors to transcription factors: potential targeting for therapeutic intervention. Leukemia.

[24] Wan C (2015). MIF4G domain containing protein regulates cell cycle and hepatic carcinogenesis by antagonizing CDK2-dependent p27 stability. Oncogene.

[25] Liu T (2017). RBFOX3 promotes tumor growth and progression via hTERT signaling and predicts a poor prognosis in hepatocellular carcinoma. Theranostics..

[26] Calvisi DF (2006). Ubiquitous activation of Ras and Jak/Stat pathways in human HCC. Gastroenterology.

[27] Newell P (2009). Ras pathway activation in hepatocellular carcinoma and anti-tumoral effect of combined sorafenib and rapamycin in vivo. J Hepatol.

[28] Dietrich P (2018). Wild type Kirsten rat sarcoma is a novel microRNA-622-regulated therapeutic target for hepatocellular carcinoma and contributes to sorafenib resistance. Gut.

[29] Lee Y (2016). MAPK Cascades in Guard Cell Signal Transduction. Front Plant Sci..

[30] Kim JY (2016). Phosphoproteomics reveals MAPK inhibitors enhance MET- and EGFR-driven AKT signaling in KRAS-mutant lung cancer. Mol Cancer Res.

[31] Wagner EF, Nebreda AR (2009). Signal integration by JNK and p38 MAPK pathways in cancer development. Nat Rev Cancer.

[32] Kondoh K, Nishida E (2007). Regulation of MAP kinases by MAP kinase phosphatases. Biochim Biophys Acta.

[33] Rasola A (2010). Activation of mitochondrial ERK protects cancer cells from death through inhibition of the permeability transition. Proc Natl Acad Sci USA..

[34] Calvisi DF (2008). Dual-specificity phosphatase 1 ubiquitination in extracellular signal-regulated kinase-mediated control of growth in human hepatocellular carcinoma. Cancer Res.

[35] Hao PP (2015). Disruption of a regulatory loop between DUSP1 and p53 contributes to hepatocellular carcinoma development and progression. J Hepatol.

[36] Muhammad N (2017). Involvement of c-Fos in the promotion of cancer stem-like cell properties in head and neck squamous cell carcinoma. Clin Cancer Res.

[37] Oishi N, Yamashita T, Kaneko S (2014). Molecular biology of liver cancer stem cells. Liver Cancer..

[38] Knoepfler PS (2008). Why myc? An unexpected ingredient in the stem cell cocktail. Cell Stem Cell.

[39] Cheng J (2018). Integrative analysis of DNA methylation and gene expression reveals hepatocellular carcinoma-specific diagnostic biomarkers. Genome Med..

[40] Beniaminov AD (2018). Deep sequencing revealed a CpG Methylation pattern associated With ALDH1L1 suppression in breast cancer. Front Genet..

